# Lung Volume Reduction in Emphysema Improves Chest Wall Asynchrony

**DOI:** 10.1378/chest.14-2380

**Published:** 2015-02-05

**Authors:** Zaid Zoumot, Antonella LoMauro, Andrea Aliverti, Christopher Nelson, Simon Ward, Simon Jordan, Michael I. Polkey, Pallav L. Shah, Nicholas S. Hopkinson

**Affiliations:** From the National Institute for Health Research Respiratory Biomedical Research Unit at the Royal Brompton and Harefield National Health Service Foundation Trust and Imperial College (Drs Zoumot, Jordan, Polkey, Shah, and Hopkinson and Messrs Nelson and Ward), London, England; the Respiratory and Critical Care Institute (Dr Zoumot), Cleveland Clinic Abu Dhabi, Abu Dhabi, United Arab Emirates; and the Dipartimento di Elettronica (Ms LoMauro and Dr Aliverti), Informazione e Bioingegneria, Politecnico di Milano, Milan, Italy.

## Abstract

**BACKGROUND:**

Lung volume reduction (LVR) techniques improve lung function in selected patients with emphysema, but the impact of LVR procedures on the asynchronous movement of different chest wall compartments, which is a feature of emphysema, is not known.

**METHODS:**

We used optoelectronic plethysmography to assess the effect of surgical and bronchoscopic LVR on chest wall asynchrony. Twenty-six patients were assessed before and 3 months after LVR (surgical [n = 9] or bronchoscopic [n = 7]) or a sham/unsuccessful bronchoscopic treatment (control subjects, n = 10). Chest wall volumes were divided into six compartments (left and right of each of pulmonary ribcage [Vrc,p], abdominal ribcage [Vrc,a], and abdomen [Vab]) and phase shift angles (θ) calculated for the asynchrony between Vrc,p and Vrc,a (θRC), and between Vrc,a and Vab (θDIA).

**RESULTS:**

Participants had an FEV_1_ of 34.6 ± 18% predicted and a residual volume of 217.8 ± 46.0% predicted with significant chest wall asynchrony during quiet breathing at baseline (θRC, 31.3° ± 38.4°; and θDIA, −38.7° ± 36.3°). Between-group difference in the change in θRC and θDIA during quiet breathing following treatment was 44.3° (95% CI, −78 to −10.6; *P* = .003) and 34.5° (95% CI, 1.4 to 67.5; *P* = .007) toward 0° (representing perfect synchrony), respectively, favoring the LVR group. Changes in θRC and θDIA were statistically significant on the treated but not the untreated sides.

**CONCLUSIONS:**

Successful LVR significantly reduces chest wall asynchrony in patients with emphysema.

In health, expansion and contraction of the ribcage and abdomen during breathing occur in tandem. During inspiration, the contracting diaphragm pushes the abdominal contents downward and the abdominal wall outward. Simultaneously, acting through its zone of apposition to the ribcage, the contacting diaphragm together with the intercostal and accessory muscles of respiration act to elevate and expand the ribcage.^1^ The ribcage has two components, which are subject to different pressures: the diaphragm-apposed part of the ribcage (abdominal ribcage compartment [RC,a]) and the upper ribcage (pulmonary ribcage [RC,p]) apposed to the visceral pleura. In COPD, lung hyperinflation means that the diaphragm is flattened and straightened, altering the angle at which it acts on RC,a. The result is mechanical distortion leading to asynchronous movement of ribcage compartments, with negative impacts on ventilatory mechanics^2,3^ as abdominal muscles are also recruited during quiet breathing. This characteristic paradoxical respiration has long been recognized clinically in COPD as the Hoover sign,^4^ although the original description is from Flint.^5^ Correlations between asynchrony of chest wall movements and the degree of airflow obstruction,^3^ breathlessness,^6^ and an earlier onset of dynamic hyperinflation during exercise^7^ have been reported.

Lung volume reduction (LVR) in patients with COPD through either surgical (LVR surgery, [LVRS]) or bronchoscopic methods seeks to correct lung hyperinflation. LVRS has been clearly shown to improve lung function, exercise capacity, and survival in selected patient groups.^8‐10^ Similarly, less invasive methods of reducing lung volume, such as bronchoscopically placed endobronchial valves and LVR coils, have also been shown to improve clinical outcomes,^11,12^ including dynamic hyperinflation.^13^ Longer-term follow-up data of patients with COPD treated with endobronchial valves suggest a survival advantage when atelectasis is successfully induced.^14,15^

The physiologic basis for benefit from LVR in emphysema was reviewed by Fessler et al,^16^ with mechanisms including increased elastic recoil and vital capacity, reduced dynamic hyperinflation, and the restoration of respiratory muscle mechanics (as the size of the treated lungs is reduced to better match the thoracic cavity and the diaphragm is restored to a more advantageous point on its length-tension relationship and a more curved configuration). In addition to these mechanisms, improvement in chest wall asynchronous movements following LVR may play a significant role. Bloch et al^17^ reported reductions in phase shift between the ribcage (as a single compartment) and the abdomen in 19 patients after LVRS. Inductive bands measuring in two dimension the lateral and anteroposterior dimensions of the ribcage and abdomen were used (RespitracePT; Non-invasive Monitoring Systems, Inc). More recently, optoelectronic plethysmography (OEP), a system that allows accurate three-dimensional (3-D) measurements of chest wall volumes, has been used to assess chest wall asynchrony in COPD in much more detail.^7,18,19^ The resultant multidimensional calculations of ribcage volumes identified asynchrony within the thorax, which is asynchronous movements between RC,a and RC,p, along with asynchrony between the abdominal compartment (Ab) and RC,a.^7,18,19^ To our knowledge, OEP has not previously been used to assess the effect of LVR on chest wall asynchrony in patients with severe COPD undergoing LVRS, and no previous studies have examined the effect of bronchoscopic LVR (BLVR) on chest wall asynchrony. We hypothesized that successful LVR, whether by LVRS or BLVR, would improve chest wall asynchrony and correlate with clinical benefit. The aims of this study, therefore, were to characterize chest wall asynchrony and assess changes therein in patients with severe COPD undergoing LVR.

## Materials and Methods

### Study Design and Participants

We recruited consecutive patients with severe COPD being assessed for LVR procedures at our institution between July 2011 and March 2013 as part of routine clinical care (unilateral LVRS^10^) or interventional BLVR trials (unilateral endobronchial valves,^20^ unilateral autologous blood instillation as a profibrotic agent^21^). All subjects had severe COPD (FEV_1_ to FVC ratio of < 0.7, and FEV_1_ of < 50% predicted) with hyperinflation (residual volume [RV] > 150% predicted). The study was approved by the National Research Ethics Committee London-Westminster (approval number 11/LO/0633). All patients provided written informed consent.

Patients enrolled in BLVR trials were randomized to have either bronchoscopy with treatment (endobronchial valves or endobronchial autologous blood instillation depending on the trial) or bronchoscopy with a sham treatment (pretend valve insertion or endobronchial instillation of normal saline). Patients who had BLVR treatment were deemed to have had successful LVR (“responders”) if there was posttreatment radiologic evidence of significant volume reduction on high-resolution CT (HRCT) scan, defined as lobar or segmental collapse with displacement of the interlobar fissures or a > 33% reduction in the size of a giant bullae on visual inspection of HRCT scan with interlobar fissure displacement and adjacent parenchyma reexpansion. In the absence of LVR following BLVR (“nonresponders”), subjects were considered to have had the equivalent of a sham bronchoscopy and their data added to sham bronchoscopy patients in a group labeled “control subjects.”

### Assessment Visits

**Figure 1 –:**
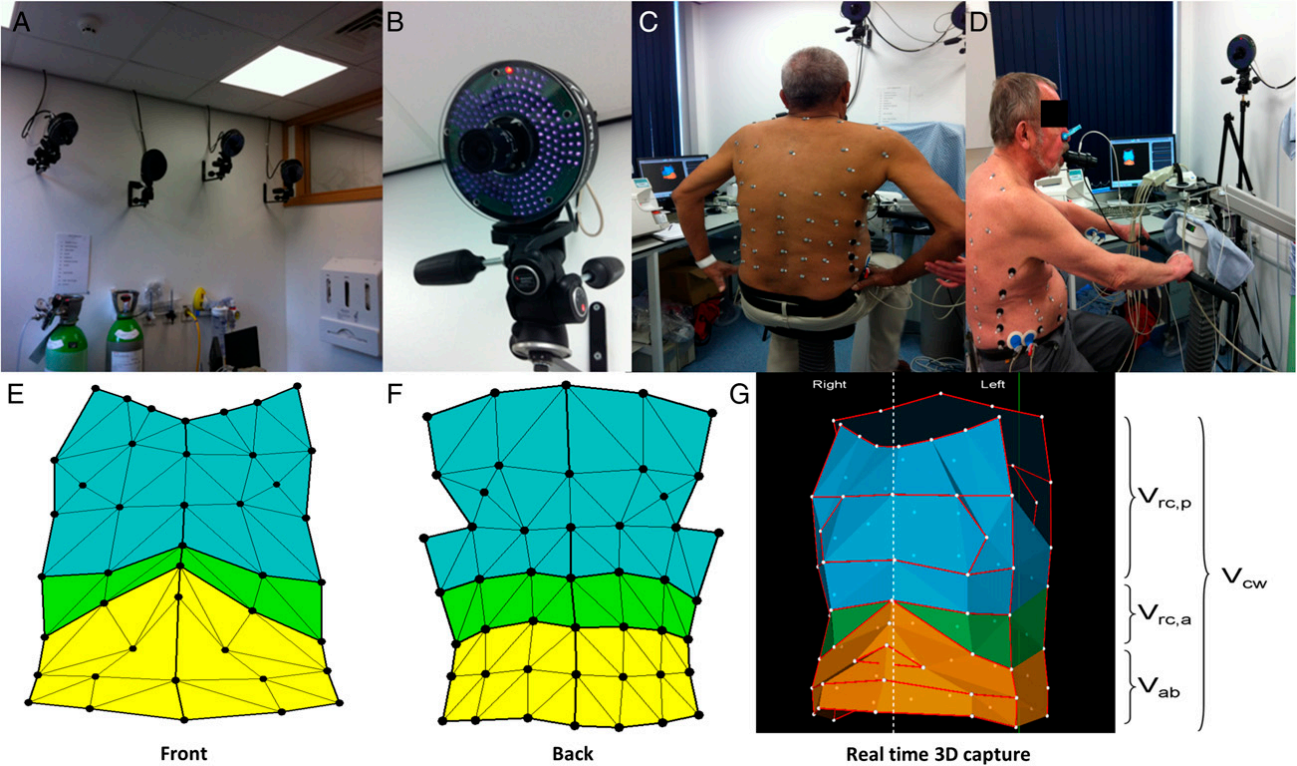
Optoelectronic plethysmography. A, B, Infrared cameras. C, D, Marker positioning. E-G, Geometric model. 3D = three-dimensional; V_ab_ = abdominal compartment volume; V_cw_ = total chest wall volume; V_rc,a_ = abdominal ribcage volume; V_rc,p_ = pulmonary ribcage volume. (The patients provided written consent for the use of the photographs.)

Baseline assessments were performed within 2 weeks prior to the surgical or bronchoscopic procedure. Data collected included demographics, pulmonary function tests (static and dynamic lung volumes and gas transfer performed as per international guidelines using the European Community of Coal and Steel Workers’ cohort normal values^22^), St. George’s Respiratory Questionnaire (SGRQ),^23^ modified Medical Research Council dyspnea score, HRCT scan of the thorax, 6-min walk distance (6MWD) per American Thoracic Society guidance,^24^ arterial blood gas analysis, and an OEP assessment. Patients also underwent endurance cycle ergometry with metabolic measurements at 75% of their maximal workload determined on an initial incremental test (performed between 4 and 24 h before the constant workload test to allow sufficient recovery time). Minute by minute inspiratory capacity (IC) maneuvers were performed to track changes in dynamic hyperinflation assessed as end-expiratory lung volume (EELV). OEP assessments comprised 5-min recordings of quiet breathing (tidal volumes) interspersed by IC maneuvers every minute, with the patient seated on a cycle ergometer at rest before the start of exercise (Fig 1). Further background and details of the OEP system used in this study can be found in e-Appendix 1. The assessments as described here were repeated 3 months after the procedure (LVR or sham).

### Data Analysis

**Figure 2 –:**
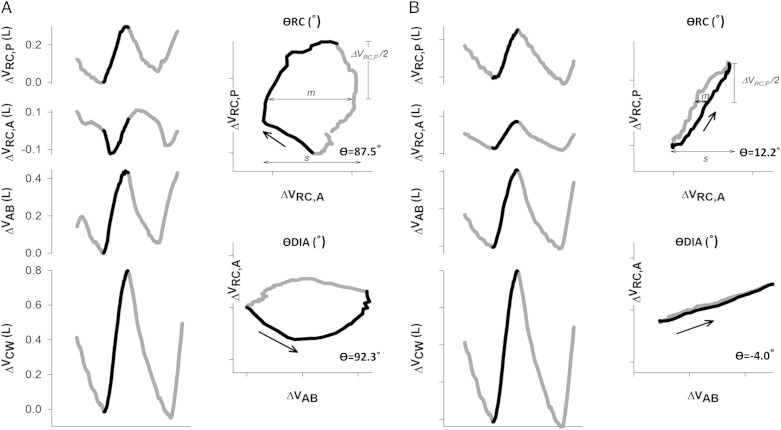
*A, B, Time courses of V_rc,p_, V_rc,a_, V_ab_, and V_cw_ during a typical respiratory cycle during quiet breathing at rest in a representative patient before (A) and after (B) lung volume reduction surgery (LVRS). Black line represents the inspiratory portion of the respiratory cycle, and asynchronous movement of abdominal ribcage compartment (RC,a) before LVRS is clearly demonstrated, as is the improvement thereof after LVRS. Lissajou figures of the dynamic loops of *Δ*V_rc,p_ vs *Δ*V_rc,a_ (*θ*RC) and *Δ*V_rc,a_ vs *Δ*V_ab_ (*θ*DIA) during quiet breathing are used to calculate *θ*. m = line parallel to the x axis at 50% of one compartment’s tidal volume; s = the second compartment’s tidal volume. Phase shift is calculated as *θ* = sin^−1^ (ms^−1^). *θ*DIA = phase shift angle between V_rc,p_ and V_rc,a_; *θ*RC = phase shift angle between V_rc,p_ and V_rc,a_. See Figure 1 legend for expansion of other abbreviations.*

A run of at least eight stable tidal breaths were used to obtain an average typical respiratory cycle during quiet breathing. This and the technically best of five IC maneuvers were used to calculate changes in chest wall volumes. Changes in total chest wall volumes during quiet breathing and IC maneuvers were split into the nine volume subdivisions: RC,p (Vrc,p), RC,a (Vrc,a), Ab (Vab), and left and right of each of Vrc,p, Vrc,a and Vab (e-Fig 1). Phase shift angles (θ) were calculated and used to assess asynchrony between various combinations of chest wall compartments^25^ (further details and representations are available in in e-Appendix 2 and Fig 2). In this system, a θ of 0° represents perfectly synchronous movement of the two compartments compared and 180° absolute asynchrony. The primary phase shift angles measured were θRC (phase shift angle between RC,p and RC,a) and θDIA (phase shift angle between RCa and Ab), including separately for treated and nontreated sides. Also measured was θRC,p, θRC,a, and θAb, each assessing the phase shift between treated and nontreated sides of the denoted compartment.

### Statistical Analysis and End Points

No previous data were available to guide a sample size calculation for this pilot study. We aimed to study eight to 10 patients undergoing LVRS, 10 to 12 patients responding to BLVR, and 20 control subjects. Analyses were performed using PASW (Predictive Analytics Software; IBM) version 20 for Windows. Data are presented as mean ± 1 SD for continuous variables. The normality test applied was the Shapiro-Wilk test. The differences between groups for continuous variables were studied using either unpaired *t* tests or the Mann-Whitney *U* test depending on the normality of their distribution, or when comparing more than one group the one-way analysis of variance with Bonferroni multiple comparison test or Kruskal-Wallis test with Dunn multiple comparison test depending on normality of distribution. Differences between groups for categorical variables were tested using the χ^2^ test. Between-group comparisons were presented as mean change with 95% CIs. Pearson *r* was used to assess correlations between θ at baseline and changes in outcome measures. For associations between changes in θ and changes in clinical outcome measures, all variables found to be univariately associated with changes in θ were entered in a stepwise binary logistic regression analysis model to identify any independent variables. A level of *P* < .05 was considered significant.

## Results

Thirty-five patients had baseline and follow-up assessments, with 26 having both baseline and 3-month postprocedure OEP studies of sufficiently good technical quality for analysis (nine LVRS, seven BLVR responders, and 10 control subjects [four sham procedures and six BLVR nonresponders]). Analysis of chest wall asynchrony requires near-perfect OEP marker tracking, in particular the compartmental borders. This was more difficult in women, in whom infrared camera visualization of markers below the breast line was often obstructed. Reasons for unsuccessful treatment in the BLVR nonresponders were as follows: one expectorated valves, one had endobronchial anatomy that precluded adequate valve placement, two had positive interlobar collateral ventilation and would thus not be expected to benefit from valve treatment, and two had no response to autologous blood LVR. These patients effectively had the equivalent of a sham bronchoscopy, and with the sham-treated patients formed the control group. Mean ± SD time between baseline and the primary follow-up assessment was 107 ± 50 days.

### Clinical Characteristics and Baseline Values

Baseline characteristics for the whole cohort and the subgroups are detailed in Table 1. There were no significant differences between any of the groups.

**TABLE 1 ]:** Baseline Values

Measure	All Subjects (N = 26)	LVRS (n = 9)	BLVR Responders (n = 7)	Control Subjects (n = 10)	*P* Value^a^
Age, y	60.6 ± 8.0	58.6 ± 9.2	60.1 ± 9.5	62.7 ± 5.8	NS
BMI, kg/m^2^	24.7 ± 4.0	23.0 ± 4.5	27.7 ± 3.3	24.2 ± 2.8	NS
Men	96	89	100	100	N/A
FEV_1_, L	1.01 ± 0.31	1.07 ± 0.39	1.08 ± 0.29	0.90 ± 0.24	NS
FEV_1_ % predicted	37.8 ± 22.7	46.4 ± 33.4	40.5 ± 14.5	28.2 ± 10.7	NS
FVC, L	3.65 ± 0.90	3.57 ± 1.18	3.94 ± 0.51	3.52 ± 0.87	NS
FVC % predicted	102.0 ± 45.6	103.0 ± 47.9	118.2 ± 41.9	89.6 ± 30.1	NS
FEV_1_/FVC	0.28 ± 0.09	0.31 ± 0.14	0.27 ± 0.06	0.26 ± 0.03	NS
RV % predicted	217.8 ± 46.0	214.3 ± 43.1	198.1 ± 41.9	234.8 ± 49.2	NS
TLC % predicted	140.3 ± 22.7	140.5 ± 31.5	139.5 ± 23.7	140.7 ± 13.1	NS
FRC % predicted	184.4 ± 25.8	181.1 ± 32.6	174.9 ± 18.3	194.0 ± 22.3	NS
Raw % predicted	328.0 ± 135.2	291.5 ± 151.6	312.7 ± 155.4	371.5 ± 103.2	NS
Tlcoc % predicted	37.4 ± 10.9	33.1 ± 11.7	41.9 ± 12.2	38.1 ± 8.1	NS
RV/TLC, %	59.0 ± 7.6	58.1 ± 7.1	56.3 ± 5.87	61.6 ± 8.83	NS
SGRQ, points	61.2 ± 13.9	59.1 ± 12.1	62.7 ± 18.0	62.0 ± 13.5	NS
mMRC dyspnea score, points	2.67 ± 0.76	2.70 ± 0.70	2.67 ± 0.52	2.60 ± 0.97	NS
6MWD, m	364.0 ± 79.2	390.6 ± 82.7	361.1 ± 63.8	342.4 ± 88.4	NS
Tlim, s	350 ± 189	340 ± 145	390 ± 130	326 ± 274	NS
EELV isotime, L	7.36 ± 1.33	7.08 ± 1.91	7.69 ± 0.66	7.61 ± 1.91	NS
Pao_2_, kPa	9.6 ± 1.2	10.0 ± 1.4	9.6 ± 1.1	9.3 ± 1.2	NS
Paco_2_, kPa	5.0 ± 0.8	5.0 ± 0.7	5.1 ± 1.1	4.8 ± 0.4	NS

Data are given as mean ± SD or %. 6MWD = 6-min walk distance; BLVR = bronchoscopic lung volume reduction; EELV = end-expiratory lung volume; FRC = functional residual capacity; LVRS = lung volume reduction surgery; mMRC = Modified Medical Research Council; N/A = not applicable; NS = not significant; Raw = airway resistance; RV = residual volume, SGRQ = St. George’s Respiratory Questionnaire; TLC = total lung capacity; Tlcoc = carbon monoxide transfer factor (corrected); Tlim = exercise time to limitation on cycle ergometer at 75% of the maximum achieved workload on a previous incremental peak exercise test.

a Kruskal-Wallis test with Dunn multiple comparison test.

### Clinical Outcomes

Successful LVR resulted in clinically and statistically significant improvements in lung function, exercise capacity, and quality of life, accompanying the radiologic evidence of volume loss. Control subjects did not exhibit any significant change in clinical outcomes (Table 2).

**TABLE 2 ]:** Change From Baseline in Clinical Outcome Measures

Measure	LVRS (n = 9)	BLVR Responders (n = 7)	All Successful LVR (n = 16)	Control Subjects (n = 10)	*P* Value^a^
ΔFEV_1_, L	0.38 ± 0.61	0.22 ± 0.22	0.31 ± 0.47	–0.01 ± 0.11	.03
FEV_1_ % change	33.6 ± 48.1	23.5 ± 20.7	29.2 ± 37.9	–2.3 ± 12.5	.03
ΔFVC, L	0.21 ± 0.82	0.57 ± 0.21	0.36 ± 0.64	–0.14 ± 0.38	.03
ΔRV, L	–0.77 ± 0.71	–1.23 ± 0.28	–0.97 ± 0.60	0.04 ± 0.48	.0001
ΔFRC, L	–0.50 ± 0.47	–0.84 ± 0.38	–0.65 ± 0.45	0.17 ± 0.45	.01
ΔTLC, L	–0.57 ± 0.58	–0.81 ± 0.42	–0.67 ± 0.52	–0.18 ± 0.36	.002
ΔRV/TLC	–6.3 ± 10.5	–9.2 ± 2.9	–7.6 ± 8.0	1.6 ± 4.3	.0005
Tlcoc, % change	10.1 ± 20.0	9.9 ± 17.6	10.0 ± 18.3	–2.1 ± 10.2	.09
ΔSGRQ, points	–15.7 ± 13.5	–12.0 ± 20.2	–14.1 ± 16.2	2.93 ± 9.76	.002
ΔmMRC, points	–0.78 ± 0.83	–0.57 ± 0.79	–0.69 ± 0.79	–0.10 ± 0.74	.12
Δ6MWD, m	30.5 ± 49.4	64.9 ± 46.8	46.5 ± 49.7	–21.3 ± 97.3	.04
ΔTlim, s	180 ± 256	157 ± 291	196 ± 23	–51.5 ± 158	.04
ΔEELV isotime, L	–0.69 ± 0.73	–1.05 ± 0.75	–0.86 ± 0.73	–0.09 ± 0.84	.03

Data are presented as mean ± SD. LVR = lung volume reduction. See Table 1 legend for expansion of other abbreviations.

a Unpaired *t* tests or Mann-Whitney test comparing the change from baseline between all LVR responders (LVRS and BLVR responders) vs control subjects. No significant difference was seen between LVRS and BLVR responder groups.

### Phase Shift Angles

There was no difference in phase shift angles between any of the groups at baseline in any of the compartments compared: θRC, θDIA, θRC treated and nontreated, θDIA treated and nontreated (Table 3), θRC,p, θRC,a, θAb. Successful LVR (LVRS and BLVR responders, n = 16) resulted in statistically significant improvements in θRC and θDIA, including θRC and θDIA during quiet breathing (Tables 4, 5). The LVR group means for θRC reduced from 37.8° ± 37.0° to 9.5° ± 17.8° (*P* = .004) and into the normal range of −18° to 18° (based on previously reported θRC values in healthy volunteers^7,19^). There are no published normal range values for θDIA. Analysis of change in phase shift angles according to treated or untreated sides of the chest wall demonstrated statistically significant improvements in θRC and θDIA on the treated side but not on the untreated side (Tables 4, 5).

**TABLE 3 ]:** Phase Shift Angles at Baseline

Phase Shift Angle	All Subjects (N = 26)	LVRS (n = 9)	BLVR Responders (n = 7)	Control Subjects (n = 10)	*P* Value^a^
Quiet breathing, TV, °					
θRC	31.3 ± 38.4	28.2 ± 31.5	50.1 ± 42.2	20.9 ± 40.3	NS
θRC treated (or worst affected) side	34.2 ± 39.9	36.2 ± 33.8	51.6 ± 4.4	20.2 ± 38.2	NS
θRC untreated side	29.6 ± 37.3	24.0 ± 30.6	46.1 ± 35.7	23.1 ± 43.6	NS
θDIA	–38.7 ± 36.3	–36.2 ± 29.7	–54.4 ± 44.7	–30.1 ± 35.4	NS
θDIA treated (or worst affected) side	–38.8 ± 36.8	–42.1 ± 31.1	–51.4 ± 45.3	–27.0 ± 35.4	NS
θDIA untreated side	–39.6 ± 36.1	–32.8 ± 28.5	–55.7 ± 42.6	–34.3 ± 37.5	NS
IC maneuver, °					
θRC	5.1 ± 58.0	9.9 ± 55.1	–25.0 ± 44.8	22.0 ± 65.2	NS
θRC treated (or worst affected) side	–12.6 ± 12.6	8.3 ± 49.8	–33.8 ± 52.6	–10.1 ± 53.8	NS
θRC untreated side	13.5 ± 58.8	9.7 ± 60.6	–8.7 ± 43.7	32.6 ± 65.2	NS
θDIA	28.9 ± 52.6	17.9 ± 15.0	65.0 ± 54.3	13.6 ± 64.1	NS
θDIA treated (or worst affected) side	23.1 ± 65.1	13.8 ± 41.4	67.1 ± 64.0	0.7 ± 73.4	NS
θDIA untreated side	23.7 ± 47.0	18.7 ± 10.1	54.8 ± 45.6	6.5 ± 59.7	NS

Data are presented as mean ± SD. IC = inspiratory capacity; θDIA = phase shift angle between abdominal ribcage compartment and abdomen compartment; θRC = phase shift angle between pulmonary ribcage and abdominal ribcage compartment; TV = tidal volumes during quiet breathing. See Table 1 legend for expansion of other abbreviations.

a Kruskal-Wallis test with Dunn multiple comparison test.

**TABLE 4 ]:** Phase Shift (θRC) During Quiet Breathing (TV) Between RC,p and RC,a

θ During Quiet Breathing	θRC, °				θRC Treated (or Worst Affected) Side, °				θRC Untreated Side, °			
	Pre	Post	Change	*P* Value^a^	Pre	Post	Change	*P* Value	Pre	Post	Change	*P* Value^a^
LVRS	28.2 ± 31.5	11.0 ± 19.1	–17.3 ± 40.5	.10	36.2 ± 33.8	10.8 ± 19.3	–25.4 ± 42.5	.13	24.0 ± 30.6	12.1 ± 21.6	–11.9 ± 41.1	.43
BLVR	50.1 ± 42.2	7.7 ± 17.1	–42.4 ± 30.3	.02	51.6 ± 47.4	2.0 ± 14.0	–49.6 ± 36.2	.02	46.1 ± 35.7	20.8 ± 35.5	–25.3 ± 47.0	.22
All LVR	37.8 ± 37.0	9.5 ± 17.8	–28.2 ± 37.5	.004	42.9 ± 39.6	6.9 ± 17.2	–36.0 ± 40.5	.005	33.6 ± 33.7	15.9 ± 27.8	–14.7 ± 42.8	.10
Control subjects	20.9 ± 40.3	36.9 ± 37.5	16.1 ± 45.1	.16	20.2 ± 38.2	38.0 ± 36.5	17.8 ± 38.1	.16	23.1 ± 43.6	35.4 ± 40.2	12.3 ± 54.9	.77

Data are presented as mean ± SD. RC,a = abdominal ribcage compartment; RC,p = pulmonary ribcage. See Table 1-3 legends for expansion of other abbreviations.

a Wilcoxon matched pairs test.

**TABLE 5 ]:** Phase Shift (θDIA) During Quiet Breathing (TV) Between RC,a and Ab

θ During Quiet Breathing	θDIA, °				θDIA Treated (or Worst Affected) Side, °				θDIA Untreated Side, °			
	Pre	Post	Change	*P* Value^a^	Pre	Post	Change	*P* Value	Pre	Post	Change	*P* Value^a^
LVRS	–36.2 ± 39.7	–7.3 ± 16.1	28.9 ± 24.1	.004	–42.1 ± 31.1	–9.3 ± 20.7	32.8 ± 22.	.004	–32.8 ± 28.5	–19.4 ± 31.2	13.5 ± 51.2	.25
BLVR	–54.4 ± 44.7	–26.4 ± 10.0	28.0 ± 38.6	.11	–51.4 ± 45.3	–21.4 ± 10.1	30.0 ± 45.3	.11	–55.7 ± 42.6	–39.4 ± 27.9	16.4 ± 44.7	.47
All LVR	–44.1 ± 36.8	–15.7 ± 16.5	28.5 ± 30.1	.002	–46.1 ± 36.9	–14.6 ± 17.6	31.6 ± 33.1	.003	–42.9 ± 36.0	–28.1 ± 30.6	14.8 ± 46.9	.16
Control subjects	–30.1 ± 35.4	–36.0 ± 36.4	–6.0 ± 51.9	.49	–27.0 ± 35.4	–38.2 ± 40.8	–11.2 ± 47.7	.32	–34.3 ± 37.5	–39.6 ± 38.6	–5.4 ± 59.1	.38

Data are presented as mean ± SD. Ab = abdominal compartment. See Table 1-4 legends for expansion of other abbreviations.

a Wilcoxon matched pairs test.

Between-group differences in the change in θ during quiet breathing were significantly different for θRC and θDIA in the direction of benefit (toward 0°), favoring patients who had successful LVR, compared with the control group. This included θRC and θDIA on the treated (but not untreated) side (Table 6). Both the LVRS and BLVR groups, when individually compared with control subjects, exhibited similar patterns of change in θRC and θDIA.

**TABLE 6 ]:** Between-Group Differences in the Change in Phase Shift Angles (θ) During Quiet Breathing Between Each of LVRS, BLVR, and All Successful LVR Groups, and the Control Group

Mean Change in θ	LVRS (n = 9)	Control Subjects (n = 10)	Between-Group Difference in Change from Baseline	*P* Value^a^	BLVR (n = 7)	Control Subjects (n = 10)	Between-Group Difference in Change from Baseline	*P* Value^a^	All Successful LVR (n = 16)	Control Subjects (n = 10)	Between-Group Difference in Mean Change From Baseline	*P* Value^a^
θRC	−17.3 ± 13.5	16.1 ± 14.3	33.3 (−75.0 to 8.4)	.03	−42.4 ± 11.5	16.1 ± 14.3	58.4 (−100.3 to −16.6)	.003	−28.2 ± 9.4	16.1 ± 14.3	44.3 (−78.0 to −10.6)	.003
θRC treated side	−25.4 ± 14.2	17.8 ± 12.0	43.2 (−82.2 to −4.3)	.04	−49.6 ± 13.7	17.8 ± 12.0	67.40 (−106.6 to −28.2)	.001	−36.0 ± 10.1	17.8 ± 12.0	53.8 (−86.8 to −20.9)	.003
θRC untreated side	−11.9 ± 13.7	−5.4 ± 18.7	6.5 (−56.4 to 43.4)	.96	−25.3 ± 17.8	−5.4 ± 18.7	19.9 (−77.3 to 37.4)	.31	−17.7 ± 10.7	−5.4 ± 18.7	12.4 (−53.6 to 28.9)	.62
θDIA	28.9 ± 8.0	−6.0 ± 16.4	34.8 (−5.1 to 74.8)	.008	28.0 ± 14.6	−6.0 ± 16.4	34.0 (−15.4 to 83.3)	.05	28.5 ± 7.5	−6.0 ± 16.4	34.5 (1.42 to 67.5)	.007
θDIA treated side	32.8 ± 7.5	−11.2 ± 15.1	44.0 (7.2 to 80.8)	.008	30.0 ± 17.1	−11.2 ± 15.1	41.2 (−7.8 to 90.3)	.07	31.6 ± 8.3	−11.2 ± 15.1	42.8 (10.2 to 75.4)	.008
θDIA untreated side	14.8 ± 11.3	−1.2 ± 4.4	16.0 (−15.7 to 47.6)	.11	16.4 ± 16.9	−1.2 ± 4.4	17.6 (−14.2 to 49.4)	.131	14.8 ± 11.3	−1.2 ± 4.4	16.0 (−15.7 to 47.6)	.13

Data are presented as No. ± SD or No. (95% CI). See Table 1-3 legends for expansion of abbreviations.

a Mann-Whitney test.

No statistically significant relationships were found between the degree of chest wall asynchrony at baseline and the magnitude of improvement in the following clinical parameters: FEV_1_, RV, RV/total lung capacity (TLC), functional residual capacity (FRC), carbon monoxide transfer factor (corrected), SGRQ, 6MWD, EELV at isotime, or exercise time to limitation on cycle ergometer at 75% of the maximum achieved workload on a previous incremental peak exercise test (Tlim) (e-Fig 2). However, there were trends toward larger benefits in some clinical outcomes in those with a greater degree of severity of chest wall asynchrony at baseline (e-Fig 2). There were no differences in θRC or θDIA during inspiratory capacity maneuvers at 3 months in any of the groups, likely because there is less asynchronous chest wall movement at baseline during these maneuvers (e-Table 1), or in the other phase shift angles measured as detailed in e-Appendix 2.

## Discussion

This is the first study, to our knowledge, to use OEP, a system integrating 3-D volume measurements from multiple markers accurately placed to delineate areas of interest on the chest wall, to demonstrate improvements in chest wall asynchrony following LVR procedures. Patients were not randomized to surgery vs BLVR, so comparisons must be approached with caution; however, responses appeared similar.

OEP demonstrated findings different from those of Bloch et al^17^ and others who used respiratory inductance plethysmography to measure asynchronous respiration, where only two measures were taken: abdominal and ribcage cross-sectional areas. The ribcage was considered a single entity, but the present OEP data demonstrate asynchrony within different ribcage compartments. Hence, the exact position of the thoracic impedance band used in the previous study, whether above or below the level of the xiphisternum or caudal to the limit of the zone of apposition of the diaphragm, could have influenced the phase shift angles measured. Bloch et al^17^ reported placing the thoracic band “within 3 cm below the nipple line,” meaning its position relative to the xiphisternum was dependent on patient height and size of the ribcage. At this level, though, the thoracic bands more likely measured what we define as the pulmonary ribcage (RC,p) in our study. Asynchronous inspiratory chest wall movement in our study, as also reported by other groups who used OEP to study patients with COPD during quiet breathing,^7,19^ was predominantly due to asynchronous movements of the RC,a (the portion of the ribcage apposed to the flattened diaphragm). RC,p and Ab did in fact move in tandem. This is consistent with a strong correlation between θRC (phase shift angle of Vrc,p in relation to Vrc,a) and θDIA (phase shift angle of Vrc,a in relation to Vab) at baseline (*r*^2^ = 0.94, *P* < .0001) (e-Fig 3). The degree of asynchrony at baseline for both θRC and θDIA correlated strongly with the degree of improvement in the same measure and in improvement in asynchrony of the other phase shift angles (e-Fig 3). Hence, the worse the asynchrony at baseline, the larger the improvement in asynchrony following LVR.

The improvements in θRC and θDIA following LVR are strongly significant on the treated side. Although improvements were seen in the nontreated side, these did not reach statistical significance, but this may relate to sample size. The finding of larger changes in chest wall asynchrony on the treated side following LVR is consistent with our knowledge of the effect lengthening the diaphragm has on respiratory mechanics. Mean θRC during quiet breathing decreased from 38.8° ± 37.0° to 9.5° ± 17.8° 3 months posttreatment (*P* < .004) in the 16 patients who had successful LVR. Using a normal range of −18° to 18° for θRC,^7,19^ nine of 16 patients had asynchronous inspiratory ribcage movements during quiet breathing at baseline and only four at 3 months postprocedure. Similarly, large improvements in θDIA were seen post-LVR (mean change of 28.5° ± 38.6° toward 0° [*P* = .002]).

Possible trends toward larger benefits in some clinical outcomes (eg, change in RV/TLC ratio and % change in FEV_1_) in those with a greater degree of severity of chest wall asynchrony at baseline can be appreciated (e-Fig 2), with correlations not reaching statistical significance in this relatively small cohort. It should be borne in mind that these patients considered for LVR and included in this study are highly selected to have severe hyperinflation. In patients with less severe hyperinflation undergoing LVR, chest wall asynchrony at baseline may be a stronger predictor of response than seen here. It has been reported that abdominal paradoxical breathing is not associated with increased dyspnea or a reduced exercise tolerance,^17,26^ and two OEP studies of exercising patients with COPD^7,19^ revealed the same, although the study by Aliverti et al^7^ demonstrated earlier dynamic hyperinflation in those with chest wall asynchrony at rest as well as increased leg fatigue during exercise (but not dyspnea) compared with those without paradoxical chest wall movements at rest.

In univariate analysis, factors associated with an improvement in θRC included improvements in static (ΔRV, ΔRV/TLC, ΔFRC) and dynamic (ΔEELV) hyperinflation as well as airways obstruction (FEV_1_). In stepwise multivariate regression analysis, reduction in FRC was retained as a factor associated with improvement in θRC (*r*^2^ = 0.41, *P* < .001) (e-Table 2). Similarly, improvement in θDIA was associated with improvements in FRC, RV/TLC, FEV_1_, SGRQ, 6MWD, and Tlim in univariate analysis with FRC retained in multivariate analysis together with Tlim, as factors associated with improvements in θDIA (*r*^2^ = 0.48, *P* = .002) (e-Table 3).

We compared the clinical responses of patients who had successful LVR who experienced improvements in θRC of > 30° or in whom θRC moved from outside to within the normal range (−18° to 18°) (n = 9), with those who did not have improvements in θRC (n = 8; either no change post-LVR or normal θRC at baseline, thus with no prospect of improvement). Improvements in clinical outcomes were almost twice as large in most parameters (Table 7). Statistical significance was achieved only for the change in RV and change in RV/TLC, although this is likely to be due to the relatively small sample size.

**TABLE 7 ]:** Change From Baseline in Clinical Outcome Measures Comparing Patients With Improvements in Ribcage Asynchrony and Those Without

Measure	θRC Improvers (n = 9)	θRC Nonimprovers (n = 7)	Between-Group Difference in Mean Change From Baseline	*P* Value^a^
% Change in FEV_1_	34.9 ± 38.7	21.9 ± 38.2	13.2 (−28.7 to 54.7)	NS
Change in RV, L	−1.24 ± 0.50	−0.62 ± 0.55	0.61 (−1.18 to −0.04)	.02
Change in RV/TLC, %	−10.9 ± 4.6	−3.4 ± 5.9	7.4 (−15.3 to 0.43)	.04
Change in FRC, L	−0.76 ± 0.44	−0.51 ± 0.47	0.26 (−0.74 to 0.23)	NS
% Change in Tlcoc	16.3 ± 20.3	2.0 ± 12.57	14.3 (−4.5 to 33.1)	NS
Change in SGRQ, points	−19.4 ± 17.6	−7.3 ± 12.2	12.0 (−28.8 to 4.8)	NS
Change in 6MWD, m	56.0 ± 45.6	32.3 ± 56.5	23.7 (33.3 to 80.7)	NS
Change in Tlim, s	283 ± 86	40 ± 89	243 (−25.2 to 512)	NS
Change in EELV isotime, L	−0.82 ± 0.32	−0.89 ± 0.21	0.08 (−0.77 to −0.93)	NS

See Table 1-3 legends for expansion of abbreviations.

a Mann Whitney test.

### Methodologic Issues

A theoretical limitation of OEP in assessing relative changes between Vrc,p and Vrc,a, previously described by Romagnoli et al,^18^ is that in patients with severe hyperinflation, the superior margin of the zone of apposition of the diaphragm to the ribcage is more caudal than normal and, therefore, the proportion of the ribcage exposed to muscles pulling in a different direction to those in contact with the upper ribcage may be smaller. The horizontal line at the level of the xiphisternum used to delineate the border between RC,p and RC,a may not exactly correspond to the true zone of apposition, which itself is likely to have shifted on the treated side following LVR. Nevertheless, Iandelli et al^27^ monitored the cephalic border of the area of apposition (ie, border between RC,p and RC,a) with ultrasound during exercise and demonstrated stability in this zone after inducing dynamic hyperinflation using a Starling resistor. In this study, we sought to identify changes in chest wall movements resulting from LVR irrespective of possible changes in the zone of apposition. Thus, if there is such a change in our cohort, it is unlikely to influence the outcomes or interpretation of data presented here. Furthermore, the very strong correlation between θRC and θDIA (where the superior margin of the zone of apposition plays no role) suggests no effect on the calculation of θRC.

Minor chest wall pain persisted in five of nine patients who had LVRS at the time of their follow-up assessment and may well have reduced chest wall movements during forced maneuvers. However, none of the patients had any pain during quiet breathing. Patients who had surgery may not have fully recovered back to their baseline levels of activity and fitness by 3 months, and a longer follow-up period may have been preferable for this reason.

## Conclusions

OEP is a novel tool that enables 3-D assessment of the mechanics of ventilation and has demonstrated improvements in chest wall asynchrony accompanied by improvements in hyperinflation following successful LVR in patients with emphysema. Successful LVR resulted in significant improvements in phase shift angles θRC (asynchrony between Vrc,p and Vrc,a) and θDIA (asynchrony between Vrc,a and Vab) at 3 months compared with baseline, and compared with control subjects, particularly on the treated side. These improvements were larger in those with the highest degrees of asynchrony at baseline and correlated with a range of clinical outcomes. Improvement in chest wall asynchrony was associated with reductions in hyperinflation, suggesting that it is linked to this important determinant of patient symptoms and outcomes. This study suggests a possible role for OEP in patient selection, helping clinicians identify those more likely to benefit from LVR improving both magnitude of benefit and responder rates from these techniques, as well as facilitating their future clinical development.

## Supplementary Material

Online Supplement

## ABBREVIATIONS

3-D: three-dimensional
6MWD: 6-min walk distance
Ab: abdominal compartment
BLVR: bronchoscopic lung volume reduction
EELV: end-expiratory lung volume
FRC: functional residual capacity
HRCT: high-resolution CT
IC: inspiratory capacity
LVR: lung volume reduction
LVRS: lung volume reduction surgery
OEP: optoelectronic plethysmography
RC,a: abdominal ribcage compartment
RC,p: pulmonary ribcage
RV: residual volume
SGRQ: St. George’s Respiratory Questionnaire
θDIA: phase shift angle between pulmonary ribcage compartmental volume and abdominal ribcage compartmental volume
θRC: phase shift angle between pulmonary ribcage compartmental volume and abdominal ribcage compartmental volume
TLC: total lung capacity
Tlim: exercise time to limitation on cycle ergometer at 75% of the maximum achieved workload on a previous incremental peak exercise test
Vab: abdominal compartmental volume
Vrc,a: abdominal ribcage compartmental volume
Vrc,p: pulmonary ribcage compartmental volume
