# Supplementary material for: Lung Volume Reduction in Emphysema Improves Chest Wall Asynchrony
Source: Chest. 2015 Feb 5;148(1):185–95. doi: 10.1378/chest.14-2380 (PMC4493874; doi:10.1378/chest.14-2380)
Supplement: Supplementary file 1 [file chest_148_1_185_ds01.pdf]

CHEST Online Supplement

Lung Volume Reduction in Emphysema Improves Chest Wall Asynchrony

Zaid Zoumot, PhD; Antonella LoMauro, MSc; Andrea Aliverti, PhD; Christopher Nelson, BSc (Hons); Simon Ward, BSc (Hons); Simon Jordan, MD; Michael I. Polkey, PhD; Pallav L. Shah, MD; and Nicholas S. Hopkinson, PhD

CHEST 2015; 148(1):185-195

e-Appendix 1:

Optoelectronic plethysmography (OEP)

The OEP system (BTS Bioengineering, Milan, Italy) utilises an automatic motion analyser to detect the positions of 89 passive markers composed of a thin film of retro-reflective paper on plastic hemispheres (5-10mm diameter).(1, 2) The markers are placed on the patient's skin using bioadhesive hypoallergenic tape following pre-defined anatomical landmarks on the chest wall (37 anterior markers, 42 posterior and 10 lateral). A horizontal line at the level of the xiphoid process delineates the boundary between RC,p and RC,a, and the costal margins anteriorly and the lowest point of the inferior costal margin posteriorly the border between RC,a and Ab.(3, 4) The midline markers over the sternum and vertebral processes delineate left from right. Eight infrared detection cameras surround the patient and non-invasively record real-time breath-by-breath images of the markers and their movement. 3-Dimentional co-ordinates of the markers are computed by stereo-photogrammetric techniques. The volume of the chest wall is calculated through the connection of points to constitute a net of tetrahedral triangles. Gauss' theorem is then used to calculate frame by frame at a sampling rate of 60 Hz the internal volume of each shape, and compartmental volumes calculated as the sum of the shapes occupying the target compartment. Therefore, OEP measurements provide both absolute chest wall volumes and changes of chest wall volume, e.g. between end-inspiration and end-expiration during quiet breathing to obtain tidal volume (TV), or between otal lung capacity (TLC) and functional residual capacity (FRC) to obtain inspiratory capacity (IC). Relative compartmental volume variations within the respiratory cycle are used to calculate asynchronies between the different compartments of the chest wall.

Online supplements are not copyedited prior to posting.

© 2015 AMERICAN COLLEGE OF CHEST PHYSICIANS. Reproduction of this article is prohibited without written permission from the American College of Chest Physicians. See online for more details. **DOI:** 10.1378/chest.14-2380

e-Appendix 2: Phase shift angle

Konno and Mead first used phase shift (ϴ) to assess rib cage and abdominal asynchrony by plotting the time courses of the change in volume of the two compartments creating a Lissajou figure (figure 2).(5) Bloch *et al.* adopted this approach when assessing change in chest wall asynchrony post LVRS, as did other groups that used OEP.(6-10) The degree of opening of the Lissajou figure corresponds to the phase shift angle (ϴ). ϴ was determined by the ratio of the distance delimited by the intercepts of the two compartmental volumes' dynamic loops on a line parallel to the x-axis at 50% of the tidal volume of the first compartmental volume (*m*), divided by the second compartmental tidal volume (*s*), as:

$$\theta = \sin^{-1}(ms^{-1})$$

In this system, a phase angle of zero° represents completely synchronous movement of the compartments and 180° absolute asynchrony. The phase shift angle ϴ was calculated separately for both quiet breathing and Inspiratory Capacity manoeuvres. In addition to asynchrony between RC,p, RC,a, and Ab, we were also interested in possible hemithoracic asynchronous chest wall movements as a result of unilateral intervention. Phase shift angles for the following compartmental combinations were then assessed, during both TV and IC manoeuvres:

- ϴRC; Phase shift angle between RC,p and RC,a.
- ϴDIA; Phase shift angle between RC,a and Ab.
- ϴRC and ϴDIA for the treated (or worst affected side in sham treated patients) and non-treated sides.
- ϴRC,p; Phase shift angle between treated and untreated sides of RC,p.
- ϴRC,a; Phase shift angle between treated and untreated sides of RC,a
- ϴAb; Phase shift angle between treated and untreated sides of Ab

Online supplements are not copyedited prior to posting.

© 2015 AMERICAN COLLEGE OF CHEST PHYSICIANS. Reproduction of this article is prohibited without written permission from the American College of Chest Physicians. See online for more details. **DOI:** 10.1378/chest.14-2380

e-Figure 1: Volume-time traces of a representative patient as measured using OEP. $V_{rc,p}$, pulmonary rib cage volume; $V_{rc,a}$, abdominal rib cage volume; V_{ab}, abdominal compartment volume; V_{cw} , total chest wall volume; $_r$ and $_l$ denote right and left.

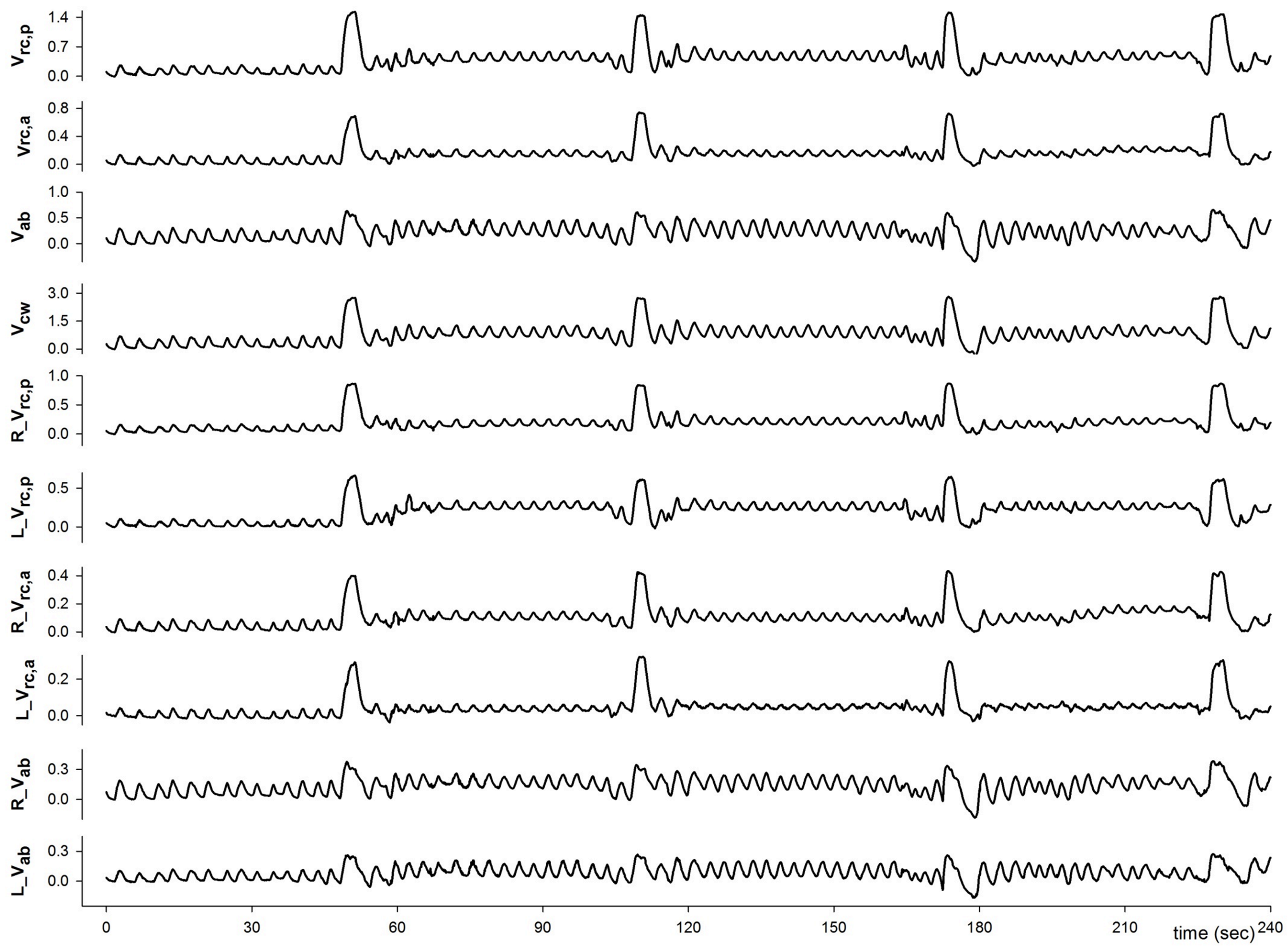

Online supplements are not copyedited prior to posting.

© 2015 AMERICAN COLLEGE OF CHEST PHYSICIANS. Reproduction of this article is prohibited without written permission from the American College of Chest Physicians. See online for more details. **DOI:** 10.1378/chest.14-2380

CHEST™ Online Supplement

e-Figure 2: The relationship between θRC at baseline and the changes in clinical outcome measures.

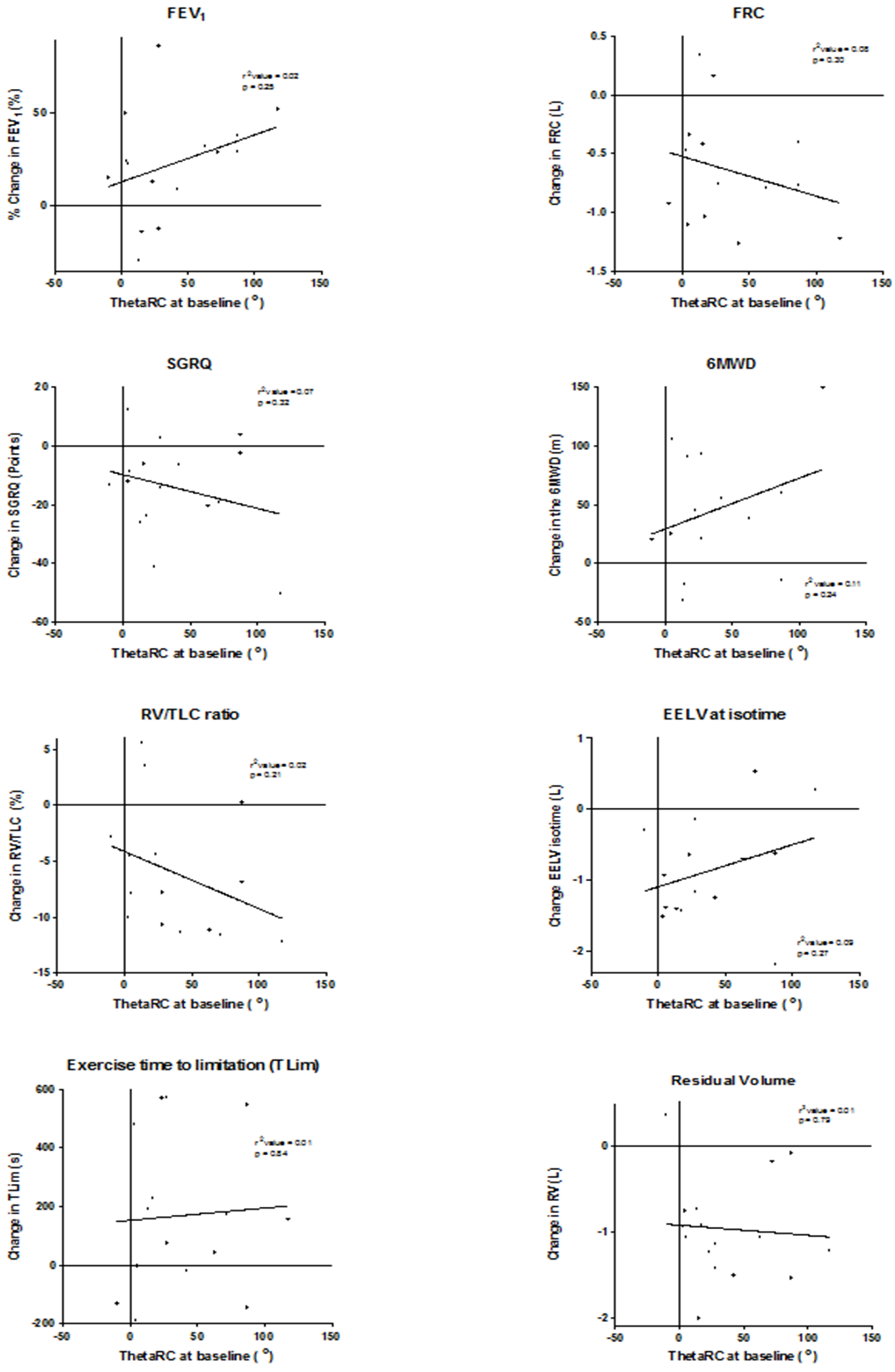

Online supplements are not copyedited prior to posting.

© 2015 AMERICAN COLLEGE OF CHEST PHYSICIANS. Reproduction of this article is prohibited without written permission from the American College of Chest Physicians. See online for more details. **DOI:** 10.1378/chest.14-2380

e-Figure 3: The relationship between phase shift at baseline and improvements in phase shift following successful lung volume reduction.

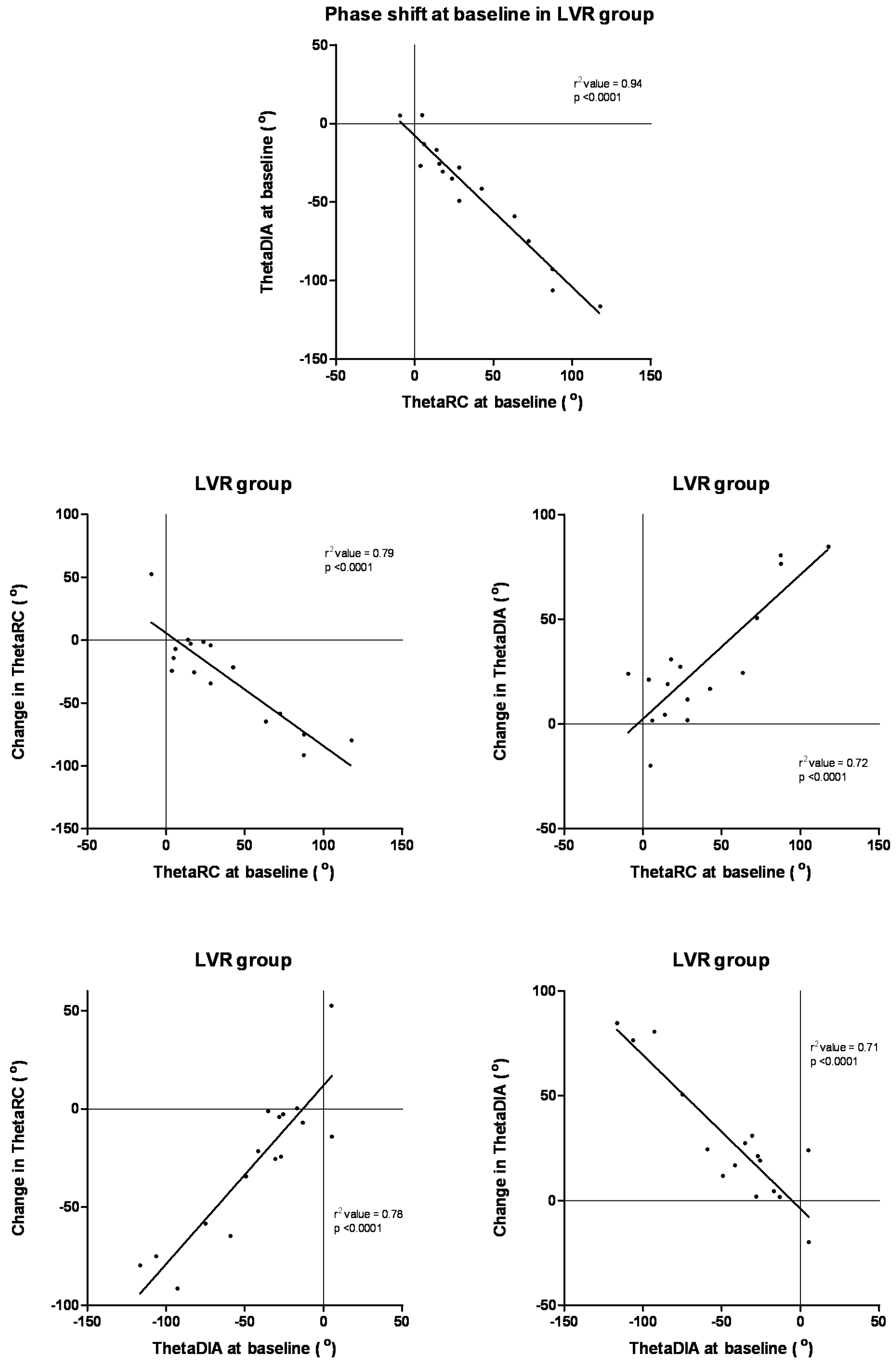

Online supplements are not copyedited prior to posting.

© 2015 AMERICAN COLLEGE OF CHEST PHYSICIANS. Reproduction of this article is prohibited without written permission from the American College of Chest Physicians. See online for more details. **DOI:** 10.1378/chest.14-2380

CHEST™ Online Supplement

e-Table 1: Phase shift during inspiratory capacity manoeuvre.

Θ during IC manoeuvre		ΘRC (°)				ΘRC Treated (or worst affected) side (°)				ΘRC Untreated side (°)			
		Pre	Post	change	p-value	Pre	Post	change	p-value	Pre	Post	change	p-value
LVRS	mean	9.9	-23.3	-33.1	0.16	8.3	-35.7	-44.0	0.16	9.7	-5.6	-15.4	0.51
	SD	55.1	42.8	52.9		49.8	58.4	59.1		60.6	36.8	67.3	
BLVR	mean	-25.0	8.4	33.4	0.11	-33.8	6.4	40.2	0.08	-8.7	11.8	20.5	0.38
	SD	44.8	14.3	42.3		52.6	12.0	50.3		43.7	21.6	36.4	
All LVR	mean	-5.4	-9.4	-4.0	0.93	-10.1	-17.3	-7.2	0.84	1.7	2.0	-0.3	0.62
	SD	52.4	36.3	58.1		53.8	48.4	68.8		53.0	31.5	57.3	
Control	mean	22.0	17.1	-4.0	0.85	-16.7	18.6	35.3	0.19	32.6	24.2	-8.3	0.92
	SD	65.2	60.2	52.2		59.8	59.5	78.1		65.2	59.8	64.6	

		ΘDIA (°)				ΘDIA Treated (or worst affected) side (°)				ΘDIA Untreated side (°)			
		Pre	Post	change	p-value	Pre	Post	change	p-value	Pre	Post	change	p-value
LVRS	mean	17.9	19.2	1.28	0.93	13.8	22.7	8.9	0.61	18.7	14.9	-3.7	0.78
	SD	15.0	48.7	46.7		41.1	54.8	50.1		10.1	37.1	41.0	
BLVR	mean	65.0	39.2	-25.8	0.61	67.0	-17.3	-84.3	0.05	54.8	40.6	-14.2	0.79
	SD	54.3	79.9	126.3		64.1	79.5	88.4		45.6	89.7	130.9	
All LVR	mean	38.5	28.0	-10.6	0.45	37.1	5.2	-31.9	0.46	34.5	26.2	-8.3	0.39
	SD	43.4	62.7	86.7		57.3	67.5	81.2		35.1	64.2	88.2	
Control	mean	13.6	5.0	-8.6	0.77	0.7	9.5	8.8	0.70	6.5	-4.7	-11.2	0.56
	SD	64.1	60.8	48.8		73.4	70.1	57.0		59.7	56.7	63.4	

Wilcoxon matched pairs test. IC, inspiratory capacity; ΘRC, phase shift angle between RC,p and RC,a; ΘDIA, phase shift angle between RC,a and Ab.

Online supplements are not copyedited prior to posting.

© 2015 AMERICAN COLLEGE OF CHEST PHYSICIANS. Reproduction of this article is prohibited without written permission from the American College of Chest Physicians. See online for more details. **DOI:** 10.1378/chest.14-2380

e-Table 2: Factors associated with change in ϴRC

Factor	Univariate regression			Multiple Stepwise Regression		
	β (95% CI)	r^2	p	β (95% CI)	r^2	p
% change in FEV_1	-0.52 (-1.04 to -0.01)	0·160	0·047			
Change in RV	29.30 (5.53 to 53.03)	0.220	0.018			
Change in RV/TLC	2.64 (0.49 to 4.79)	0.219	0.018			
Change in FRC	45.62 (20.21 to 71.04)	0.380	0.001	49.87 (22.22 to 75.53)	0.409	0.001
Change in SGRQ	0.95 (-0.19 to 2.09)	0·115	0·087			
Change in 6MWD	-0.19 (-0.45 to 0.73)	0·097	0·149			
Change in Tlim	-0.07 (-0.15 to 0.14)	0.128	0.097			
Change in EELV isotime	24.51 (2.43 to 46.72)	0·201	0.032			

Online supplements are not copyedited prior to posting.

© 2015 AMERICAN COLLEGE OF CHEST PHYSICIANS. Reproduction of this article is prohibited without written permission from the American College of Chest Physicians. See online for more details. **DOI:** 10.1378/chest.14-2380

e-Table 3: Factors associated with change in ΘDIA

Factor	Univariate regression			Multiple Stepwise Regression		
	β (95% CI)	r^2	p	β (95% CI)	r^2	p
% change in FEV_1	0.48 (0.04 to 0.91)	0.180	0.034		0.475	
Change in RV	-20.36 (-41.61 to 0.89)	0.146	0.060			
Change in RV/TLC	-2.08 (-3.96 to -0.20)	0.186	0.031			
Change in FRC	-36.24 (-58.84 to -13.65)	0.324	0.003	-40.46 (-65.74 to -15.19)		0.002
Change in SGRQ	-1.23 (-2.12 to -0.34)	0.262	0.009			
Change in 6MWD	0.23 (0.02 to 0.44)	0.202	0.032			
Change in Tlim	0.07 (0.01 to 0.14)	0.211	0.031	0.07 (0.01 to 0.12)		0.018
Change in EELV isotime	-17.65 (-37.22 to 1.93)	0.143	0.075			

Online supplements are not copyedited prior to posting.

© 2015 AMERICAN COLLEGE OF CHEST PHYSICIANS. Reproduction of this article is prohibited without written permission from the American College of Chest Physicians. See online for more details. **DOI:** 10.1378/chest.14-2380
